# Assessment of an effective quantitative model with multi-criteria decision-making method for sustainable campus

**DOI:** 10.1007/s11356-024-32040-7

**Published:** 2024-01-20

**Authors:** Onur Aksoy, Sara Demir, Nazli Deniz Ersoz, Merve Dilman Gokkaya

**Affiliations:** Landscape Architecture Department, Forest Faculty, Bursa Technical University, Bursa, Turkey

**Keywords:** Sustainable campus criteria, Climate change, Multi-criteria decision-making (MCDM) method, Analytic hierarchy process (AHP), Planting and structural campus components

## Abstract

Sustainability is a current topic in public open green spaces such as university campuses. In order to ensure the sustainability of the campus areas, it is necessary to determine the criteria for the sustainable campus landscape. Bursa Uludağ University Gorukle Campus in Bursa was chosen as the study area in this research. The aim of the study is to identify suitable sustainable campus criteria with a focus on landscape and to prioritize appropriate sustainable campus strategies determined according to these criteria. In this context, first, field studies and literature research were carried out. Second, sustainable campus criteria were classified as criteria and section. The section was then divided into credit. All these credits were ranked according to their priorities. Analytic hierarchy process, one of the multi-criteria decision-making methods, was used while ranking. According to the result of the criteria, planting landscape components were determined to be more important than structural landscape components. Among the section, the transport category was found as the highest priority criterion. The use of vegetable wastes as compost was also determined as the most important criterion among all credits. The method and findings of this research may set an example for determining priorities of the sustainable campus criteria in Turkey and developing countries with a participatory management approach.

## Introduction

The concept of sustainability has been on the agenda of the world since the 1970s. However, the formation of a conceptual framework took place after the 1980s. The concept of sustainability was defined in the Brundtland Report in 1987 (Ozdemir et al. 2020). The concept has been reinterpreted as social, economic, and environmental development over time (Zhu et al. 2022). In addition to fields such as industry, economy, and urbanization, it has also come to the fore in universities and also public and private sectors (Ozdemir et al. 2020). Since the Stockholm Declaration in 1972, studies on sustainability have started and university institutions have been involved in studies in this direction since 1990 (Saygin and Ulusoy 2011). This has led to the design of university campuses taking into account the principle of sustainability (Patel and Patel 2012).

Universities have an important role in social, economic, and political life. These important various roles make universities one of the important key models in ensuring sustainable development (Kalayci Onac et al. 2021). A university is often evaluated to a miniature city model, since it consists almost all the city functions. Environmental resources are important for cities (Li et al. 2018). The planning and management of the university campus with the social and physical infrastructure affects the development of cities in the urban context. The university campus attracts attention with its feature of being not only a view located in the urban area, but also an urban area that forms an integral part of the city. Accordingly, the physical settlement situation; the quality of its design; definition of its functional program; and consequently its governance may influence particular relationships with the city (Magdaniel 2013). The concept of a sustainable campus often emphasizes reducing costs, recycling materials, saving energy, and encouraging people to behave “greenly” (Li et al. 2018). The main purpose of sustainable design in campus areas is to mitigate the consumption of basic resources such as energy, water, and raw materials (Patel and Patel 2012).

The student and staff activities of the university campus cause significant electricity consumption. To achieve this goal, campuses must effectively reduce energy consumption in their daily operations. An ideal solution is to use green systems to generate electricity. Such systems can sometimes meet 80% of the cooling and heating energy needs (Li et al. 2018). Reducing energy consumption is critical to campus sustainability. For example, school buildings account for 13% of all building energy consumption in the USA (Chen et al. 2018). In addition, universities are encouraged to use renewable energy by government departments due to their high energy consumption (Saygin and Ulusoy 2011). Kashan University is one of the leading universities in designing and building renewable power plants in Iran. The university can meet more than 70% of its energy consumption (Monemzadeh and Talebi-Dastenaei 2021).

Another important criterion in sustainable campus criteria is water efficiency. Sustainability of water in campus areas mainly hinges on reducing consumption, collecting water, and recycling water (Amr et al. 2016). Applications to be made for sustainable water efficiency in campus areas; rain gardens, permeable flooring, green roof, natural plant use, and gray water use; and the use of effective irrigation systems cover the use (Patel and Patel 2012). As an example, Birkenfeld Campus is known as Germany’s greenest campus and Europe’s first zero-emission campus (Helling and Bölsche 2021).

Another criterion that is essential to be examined in sustainable university campuses is transportation. In university campuses designed with sustainable transportation in mind, issues such as reducing vehicle use, promoting public transport, shared car use, and fuel-efficient vehicles are recommended. Ali et al. (2018) emphasize the importance of accessing universities by public transportation. In addition, bicycle paths and bicycle parks should be designed within the transportation circulation in the campus areas. For example, the University of Arizona has made the campus bike-friendly with more than 11,000 bike parking spaces and secure bike paths (Finlay and Massey 2012). In another example, The IPB Dramaga Campus has secure bike paths, shower facilities, lockers, and a bike repair shop for cyclists (Sisriany and Fatimah 2017).

Another important criterion in sustainable campus criteria is material and recycling. Many experts related to the ecosystem state that rational waste treatment is very important for universities. Clearly, recycling materials and waste can reduce solid waste pollution and reduce purchasing needs (Li et al. 2018). Recycling of solid waste is important as it may reduce the negative effects on the environment (Saygin and Ulusoy 2011). In this context, solid waste and vegetable wastes are recycled in university campuses (Patel and Patel 2012); and while suitable solid wastes are reused as urban features (Mendoza et al. 2019), plant material is used as compost (Kalayci Onac et al. 2021). For example, the University of Nottingham has made a significant reduction in waste per student in recycling. In this context, it has saved 11,000 tons of C (Sivapalan et al. 2016).

In addition, increasing open green spaces in university campuses is important in terms of sustainability and ecology of the campus. The green vegetation of campuses makes universities more sustainable as it not only provides views but can also be used as an ecological drainage system to reduce the area of impervious surfaces and reduce temperature and CO_2_ emissions (Li et al. 2018). In campus areas with increasing green areas, reducing the UHI effect and CO_2_ emissions (Chen and You 2020) can provide habitat areas for wildlife.

In a sustainable campus model, providing internationally determined standards and developing a management and monitoring model in accordance with these standards is of great importance for a healthy, effective, and interactive campus. The concept of sustainability on campuses in Turkey is completely new. In fact, there is no campus planned and designed in full compliance with international standards or criteria. In this research, Bursa Uludag University (BUU) Gorukle Campus located in Bursa province, which is under pressure with the urban texture of the western, eastern, and northern parts, but still preserves its green texture, was determined as the study area. Gorukle Campus is the 5th largest campus in Turkey. Many applications have been carried out within the scope of sustainable campus in Gorukle Campus. However, despite all these practices, the campus was able to take its place in the 335th place in the GreenMetric classification in 2021. Many practices and recommendations have been developed regarding planning, design, and implementation for sustainable campuses. However, in these studies, the “landscape” factor, which concerns the open and green spaces of campus, remained weaker than architectural and construction application. This study evaluated the sustainable design and planning studies proposed for the campus and its immediate surroundings, mainly from the landscape aspect. In this context, the AHP method, which is one of the multi-criteria decision-making (MCDM) methods and widely used, was designed to evaluate sustainable campuses within the scope of landscape architecture planning, design, and application. The evaluation made is rare in that it uses AHP from the perspective of planning, design, and application to be proposed within the scope of landscape. In this context, the factors related to the landscape are ranked according to their importance with stakeholders and experts objectively. Besides this research, it is aimed to develop a digitized sustainable campus model that offers structural and plant solutions specific to each campus, within the scope of sustainable landscaping practices planned on campuses. Campus criteria, which consist of solution proposals in accordance with international standards, were determined and each criterion was ranked according to their priority with a participatory management approach. AHP, which is one of the MCDM methods and has high accuracy, was used in ordering these criteria. In the AHP analysis, the criteria were compared, and appropriate planting and structural landscape planning and design strategies were proposed for a sustainable campus. Some studies have already been carried out on sustainable campus. However, this study is the first to evaluate AHP analysis, one of the participatory management approaches and MCDM methods, within the scope of landscape planning, design, and applications on campuses in Turkey. Unlike other sustainable campus studies, this research took the participatory management approach into account for the first time in Turkey. The methods and outputs of this study, which supports the participatory management approach, can set an example for the development of a sustainable campus model in accordance with international standards in campuses in Turkey and other developing countries.

## Material and method

### Material: Study area

**Fig. 1:**
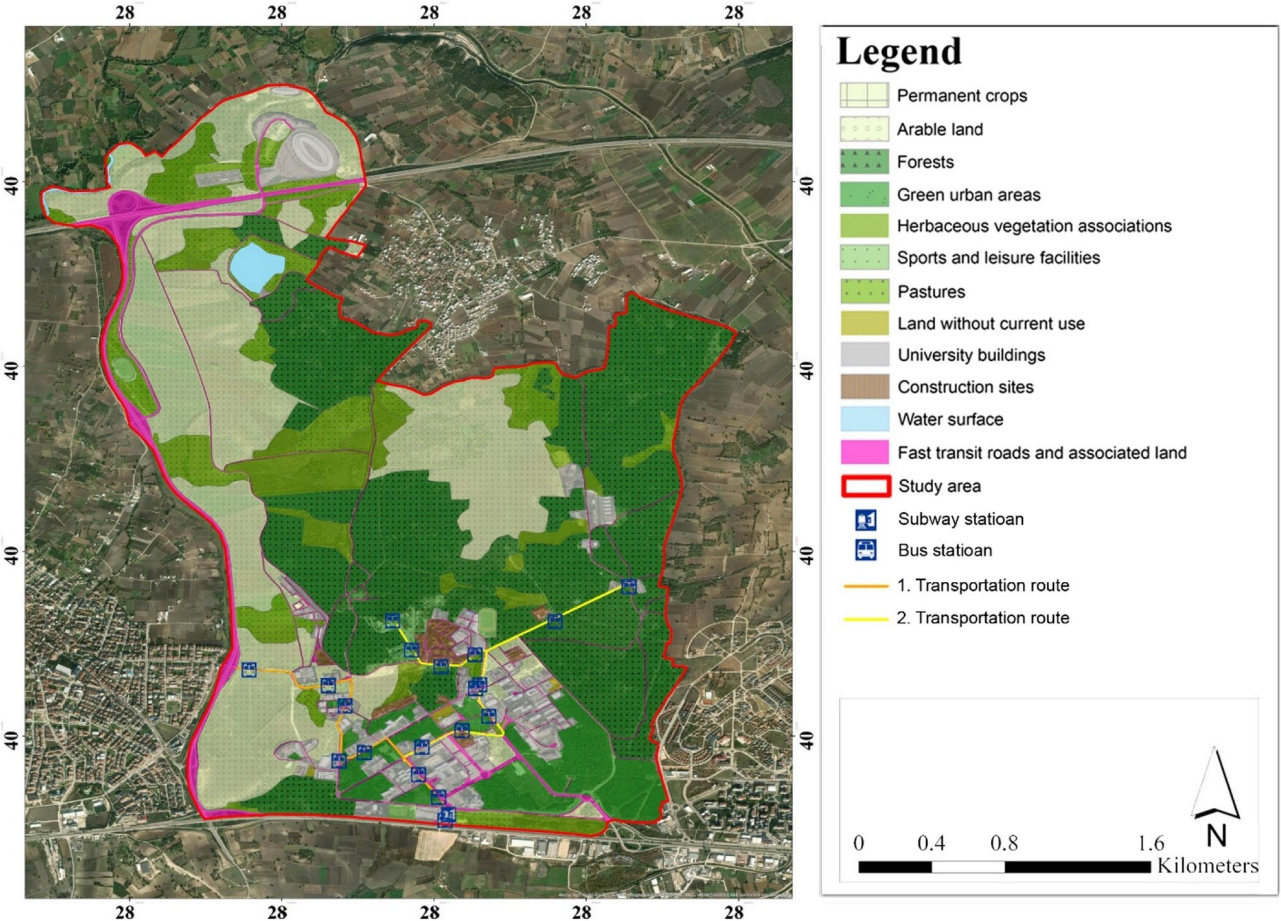
Study area, land use, and current transportation status of the area

The study area constitutes the main material of the research. In this context, BUU Gorukle Campus, located in the Nilüfer district of Bursa province in Turkey, was determined as the study area (Fig. 1). Located in an urban area, the university campus consists of an area of 1212 ha. It consists of university buildings, lodging, dormitory buildings, hospital, cafeteria, forest areas, water surface, and agricultural areas. This area has 460 ha forest area, 339 ha agricultural area, 141 ha structured area, 89 ha pasture area, 90 ha open green area, 69 ha natural vegetative land, and 8 ha water surface, and the remaining areas consist of orchards and vacant area (UUCWTD 2022). 51.07% of these areas consist of open green areas, which plays an effective role in reducing CO_2_ emissions. Parking lots are areas where the UHI effect is seen intensely. Besides, the amount of green space per capita in the campus area was calculated by dividing the total green area by the population on the campus. The amount of green space per person is 112 m^2^. The parking areas within the structural areas in the study area cover an area of 9.3 ha. The entrance of the campus area is provided from two different points. Two bus lines are actively working in the area. Thirty-eight bus stops were counted in line with the investigations made in the area (Fig. 1). In addition to bus transportation, a metro station passes through the study area.

In the study area, 4 bicycle parking lots were built by Nilüfer Municipality in 2022. However, in line with the investigations in the field studies, it is not possible to reach every point of the area by bicycle. In addition, it has been determined that bicycle paths are not defined in many places. In the study conducted by Sevimli (2021), BUU Gorukle Campus consists of 11.4 ha impermeable surfaces such as asphalt and concrete pavement. Regarding the hydrological structure of the area, there is a wide water surface of 8 ha in the north of the area. In addition, a branch of the Nilüfer Stream passes in the northwest of the area.

There are many applications planned to be implemented within the scope of waste management at BUU Gorukle Campus. The first of these is to carry out educational activities for students and staff in order to prevent waste generation. Another application is the planning of investments to prevent waste generation and the implementation of the zero waste project in all its dimensions. Finally, it has been planned to establish management models for all types of waste (domestic, electronic, hazardous, medical, food-borne, excavation, etc.) generated on the campus (BUU 2022a). There is no application in the field in terms of renewable energy. However, it is planned to install solar energy panels in the BUU Hospital parking lot and its immediate surroundings (BUU 2022b).

### Method

The study consists of four stages. First, literature research was conducted and the current situation was assessed. Within the scope of the literature data examined later, criteria and sections for sustainable campuses were determined. The AHP method, which is one of the MCDM methods, was used while determining criteria and sections and the weight scores of credits under sections. Within the scope of the analyses obtained in the last stage, the strategies required for sustainable campus planning at BUU Gorukle Campus were evaluated. Each step is explained in detail below.

#### Determination of the current situation

At this stage, demographic, natural, and structural features of BUU Gorukle Campus were examined. The natural and structural data of the area were collected and analyzed in the geographic information system (GIS) environment. According to the last census in 2021, there are a total of 3869 people on the campus, of whom 2074 are academic staff and 1795 are university staff. The total campus population, including students, is 55,065 (Altun 2022). Then, the natural data in the field were examined. Considering Turkey’s digital elevation model (DEM) data, elevation, aspect, and slope maps were created in the ArcGIS environment. In addition, the map showing the soil status of the area has been digitized taking into account the Turkey Soil Atlas (TSA 2022). The map showing the land use in the study area was obtained from CORINE (2018) and UUCWTD (2022) data. All these topographic data were evaluated to specify the sustainable campus criteria.

#### Identification of sustainable campus criteria

Within the scope of the study, the definition of sustainability, examples of sustainable practices made in campuses, and fieldwork and visual field analyses were examined while determining the sustainability criteria in BUU Gorukle Campus. In addition, examples from the world and Turkey, which are characterized as green certification systems (CASBEE-UD, LEED-ND, BREEAM, Green Metric, etc.) and sustainable campuses, and the practices recommended within the scope of sustainability have also been taken into account. In this study, many of the practices suggested under “section and credit” are taken from the titles of certification systems such as LEED-ND, BREEAM Communities, CASBEE-UD, and DGNB-NSQ, which are green certification systems (Table 1). These certification systems certify many areas such as schools, hospitals, new buildings, neighborhoods, and campuses. For example, in the transportation section, credits such as “public transportation access, fuel-efficient vehicles, reducing parking lot capacity, bicycle road” and LEED BD + C and BREEAM Communities certification systems were taken into consideration. In addition, some of the “gray water use, natural material use etc. credit” systems were taken from the certification system such as DGNB. Credit titles such as increasing green spaces, urban furniture, use of certified recycled wood, and reducing the UHI effect were taken from the CASBEE certification system. Apart from certification systems, many sustainable practices recommended for campus areas such as “using plant wastes as compost, road afforestation, tree shapes, and placements, etc.” are listed under “credits” in Table 1. Most of these applications were obtained as a result of examinations within the scope of the literature studies. While determining the criteria, structural and planting component to be evaluated within the scope of the study were determined as the criteria. Local environment, water, energy, waste, and transportation were also determined as sections under structural and planting component. The criteria for the sustainable campuses proposed to BUU Gorukle Campus within the scope of the study are shown in Table 1.

**Table 1:** Criteria for sustainable campuses proposed to BUU Gorukle Campus

Criteria	Section	Credit	References
A: Planting landscape component	A1: Local environment	A.1.1: Reducing the UHI effect	(CASBEE 2023; Chen and You 2020)
		A.1.2: CO_2_ emission reduction	(Chen and You 2020)
		A.1.3: Development outside protected areas (site selection)	(UGB Council 2009)
		A.1.4: Increasing green spaces	(Chen and You 2020; UGB Council 2009; CASBEE 2023)
	A2: Water efficiency	A.2.1: Rain garden	(Song 2022)
		A.2.2: Xeriscape garden	(Hilaire et al. 2008)
		A.2.3: Green roof	(Amr et al. 2016; UGB Council 2009)
		A.2.4: Vegetation swales and bioswales	(Amr et al. 2016)
		A.2.5: Natural planting	(Amr et al. 2016)
		A.2.6: Reducing grass areas	(Vickers 2006)
	A3: Energy efficiency	A.3.1: Vertical garden	(Perez et al. 2011)
		A.3.2: Green roof	(Kalayci Onac et al. 2021; UGB Council 2009)
		A.3.3: Wind curtain	(Zhu et al. 2022)
	A4: Waste management	A.4.1: Using plant wastes as compost	(Cano et al. 2023)
		A.4.2: Use of plant waste as mulch	(Fitzgerald and Ries 1997)
	A5: Transport	A.5.1:Road afforestation	(Lachapelle et al. 2023)
		A.5.2: Tree shapes and placements	(Lachapelle et al. 2023)
B: Structural landscape component	B1: Local environment	B.1.1:Use of regional flooring materials	(DGNB 2023; UGB Council 2009)
		B.1.2:Construction activity pollution prevention	(UGB Council 2009)
		B.1.3:Use of certified wood	(UGB Council 2009; CASBEE 2023)
		B.1.4:Use of rapidly renewable materials	(UGB Council 2009)
	B2: Water efficiency	B.2.1:Graywateruse	(Boano et al. 2020; DGNB 2023; BREEAM 2022; CASBEE 2023)
		B.2.2:Storage of rainwater	(BREEAM 2022; UGB Council 2009; CASBEE 2023)
		B.2.3:Use of permeable flooring material	(Guan et al. 2021)
	B3: Energy efficiency	B.3.1:Use of solar panel	(Kalayci Onac et al. 2021; Li et al. 2018)
		B.3.2:Urban furniture that produces their own energy	(Ermiş and Karatekin 2019)
	B4: Waste management	B.4.1:Recycled urban furniture	(Kurtaslan 2020; CASBEE 2023)
		B.4.2:Use of recycled flooring material	(Gayarre et al. 2017)
		B.4.3:On-site recycling of wastes such as paper, metal, and glass	(Kurtaslan 2020; Li et al. 2018)
	B5: Transport	B.5.1:Public transportation access	(BREEAM 2022; Kalayci Onac et al. 2021; UGB Council 2009; Ali et al. 2018)
		B.5.2:Bicycle road	
		B.5.3:Fuel efficient vehicles	
		B.5.4: Reducing parking lot capacity	

#### Quantification campus criteria with AHP

**Fig. 2:**
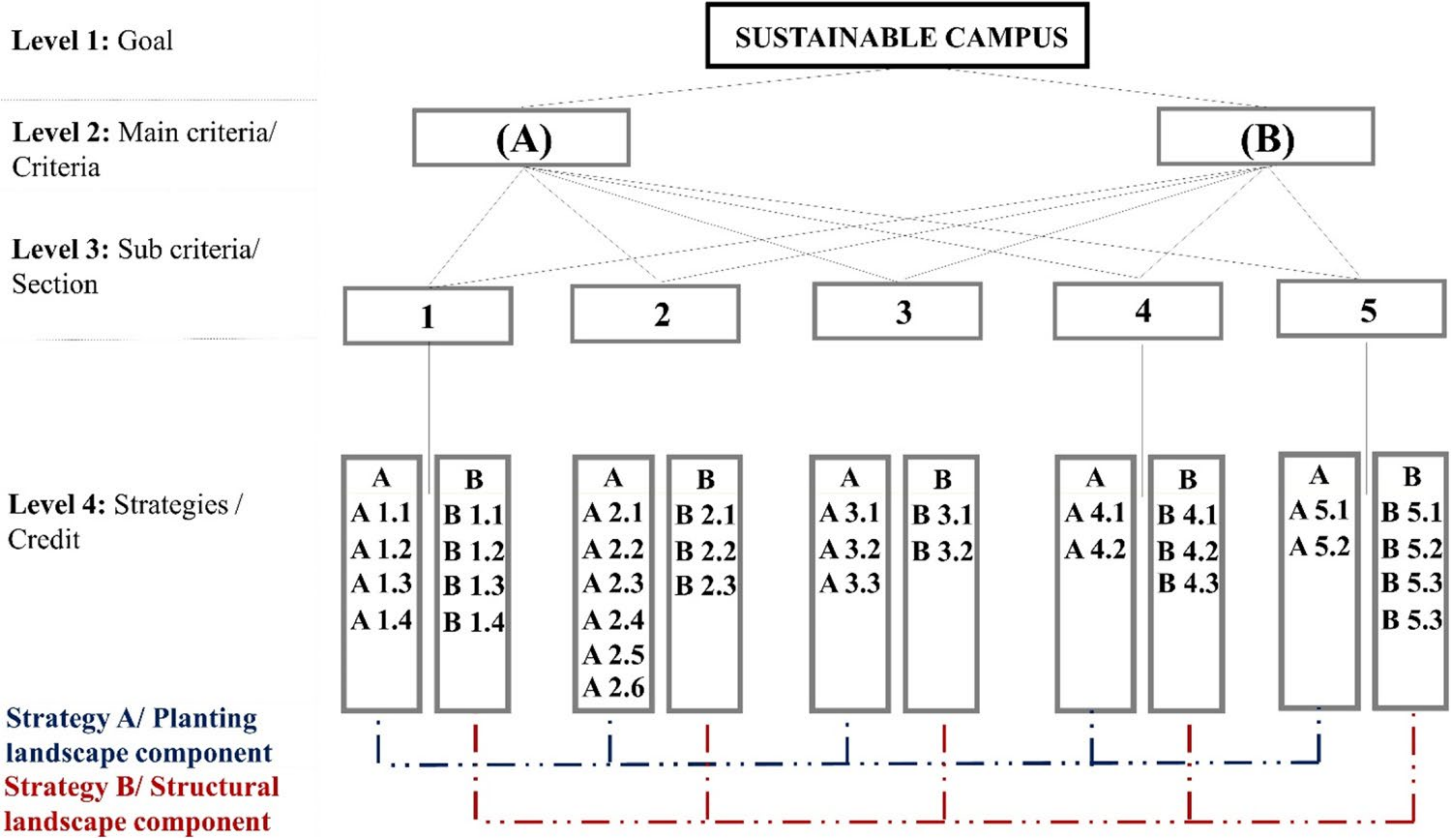
Hierarchical 4-level structure of the proposed AHP decision-making model for measuring the sustainability campus

Introduced by Saaty, AHP is one of the most common MCDM methods. Applying this principle, Saaty (1980) and Saaty (1982) developed a comparison method that models a hierarchical decision problem framework that includes various criteria with one-way relationships (Carli et al. 2018). Synthetically, the AHP methodology involves the following four steps: structuring the decision problem into a hierarchical model (1), developing pairwise comparisons and obtaining the judgmental matrices (2), determining the local priorities and consistency of comparisons (3), and determining the final priorities (Carli et al. 2018) (Fig. 2).

AHP constitutes a systematic approach to evaluate and integrate the effects of different factors, including some levels for qualitative and quantitative information (Perçin 2006). It has been determined that AHP analysis is an appropriate approach to determine the weights of the selection criteria in such a selection problem that aims to compare more than one criterion objectively (Chen et al. 2018). “Weight value” is the weights obtained according to the answers given by the users of each criterion, section, and credit as a result of the survey directed to campus users and experts within the scope of AHP. This weighting system was obtained thanks to the formulas entered into Microsoft Office Excel (2010) and Expert Choice 11 software. In this context, AHP analysis was applied by using all sustainable campus criteria in the decision hierarchy. In order to evaluate the sustainability criteria in Gorukle Campus, criteria and sections were established. Credits were created in order to evaluate sections within themselves and to increase the originality of the study. The AHP was utilized due to its simple, flexible, and quantitative structure. This method also supports the participatory management approach and objectively lists the priorities in the decision-making process (Carli et al. 2018). For these reasons, it has been considered as an appropriate method in the evaluation of sustainable campus in Gorukle Campus.

A questionnaire was conducted to quantify the AHP analysis with the participation of university students, staff, and experts. Works of Saaty (1990) and Carli et al. (2018) were used for this purpose. In the sampling calculation, the reliability rate was found to be 95%. It was also calculated that 382 people should be surveyed and 390 people were surveyed. A questionnaire form was prepared in which the sustainable campus criteria determined within the scope of the study were compared with each other in pairs. In the questionnaire, all sustainability criteria in the decision hierarchy were questioned under the main components of the AHP analysis to determine the sustainability potential of Gorukle Campus.

**Fig. 3:**
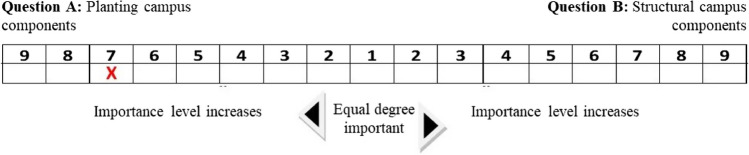
The criteria marked by one of the users for the 1st question in the survey

While preparing the questionnaire, the two decision elements (A and B) given to each question were compared with each other. According to the given questionnaire, the users were asked “Choose the decision element that is more important in your opinion” in terms of the features and functions of the criteria, sections, and credits. Users were asked to rate the questions based on their importance from 1 to 9. For example, in Fig. 3, the first survey question, which was asked to all users on campus, was “Are vegetative landscape components or structural landscape components more important in campus areas?” as shown in Fig. 3, one of the users observed that vegetative landscape components are 7 times more important than structural landscape components.

While preparing the questionnaire, within the scope of the literature research, the question was asked which of the vegetal and structural landscape components shown in Table 1 and under the title of “criteria” category is more important. Then, based on the questionnaire answers, the result of which criterion for question 1 is more important will be revealed. The result will determine the score weight of the categories in the “section” in Table 1. Then, 10 questions were asked about which of the 5 headings in the “section” category was more important. The weight of each “section” was determined according to the questions asked. Then, this process was repeated for each “credit” heading in Table 1 and the priority weighting regarding the application and design in the sustainable campuses intended to be built within the scope of the study was determined. As a result of the study, weight scores for the criteria, section, and credit headings shown in Table 1 were determined. The studies on implementation and design in sustainable campuses (Table 1) were created by taking into consideration the literature research and the practices used in CASBEE-UD, LEED-ND, BREEAM Communities, DGNB-NSQ, etc. certificates. There are 56 questions in total in the survey by pairwise comparison. The clarity of the questionnaire is one of the most important factors that increase the consistency ratio (CTR). For this reason, while the questionnaire was applied to students and university staff for the main and sections, credits were answered by experts in the field, since they contain expertise. In order to determine the reliability and consistency of the questionnaire, a preliminary assessment survey was applied by interviewing the students, university staff, and academic staff using the campus. In order to evaluate criteria and sections, a total of 200 students and 100 university staff were asked questionnaires. The questionnaire study, which was directed to the related expert groups working on the subject, was carried out face to face. A total of 90 experts (landscape architects, architects, city-regional planners, and civil engineers) were interviewed. Individual questionnaire data were organized in Microsoft Office Excel (2010) software and geometric mean of each item of the questionnaire was calculated by the Expert Choice11 software. According to Carli et al. (2018), the consistency ratio has to be less than 0.10 for acceptability of the study.

#### Planning and design proposals

The last phase of this study, which was carried out in line with the opinions of university users and experts and took into account the priorities in quantitative AHP analysis, offered structural and planting suggestions for sustainable campuses that could be referenced in other campuses.

## Results

### Prioritization criteria of sustainable campus

**Fig. 4:**
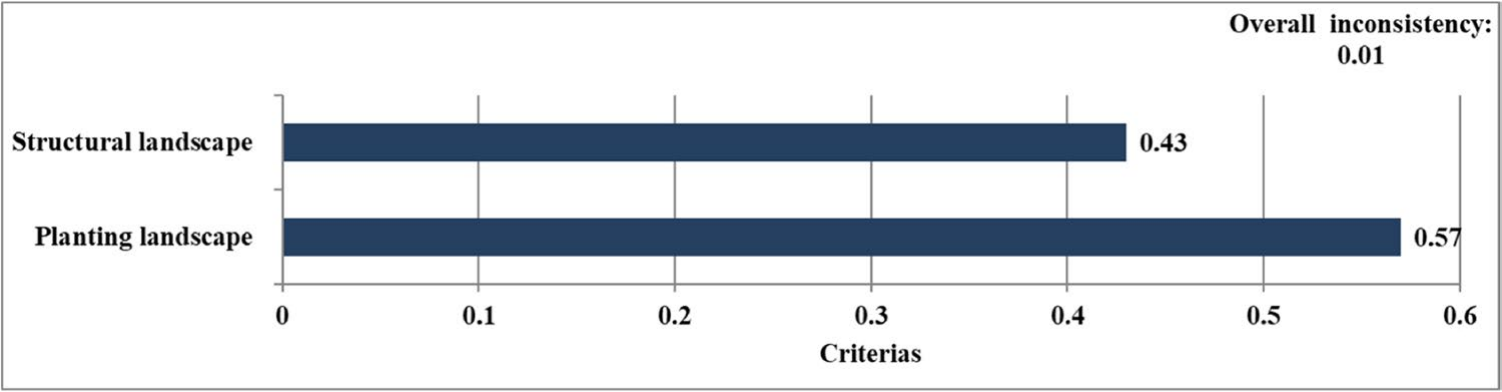
Prioritizing the structural and planting sustainable campus criteria

In this study, all criteria were ranked according to their priorities. According to the results, the ranking between criteria was respectively A: planting landscape elements (0.57) and B: structural landscape elements (0.43) (Fig. 4). The CTR ratio among criteria was calculated as “0.01”.

### Prioritization sections of sustainable campus

**Fig. 5:**
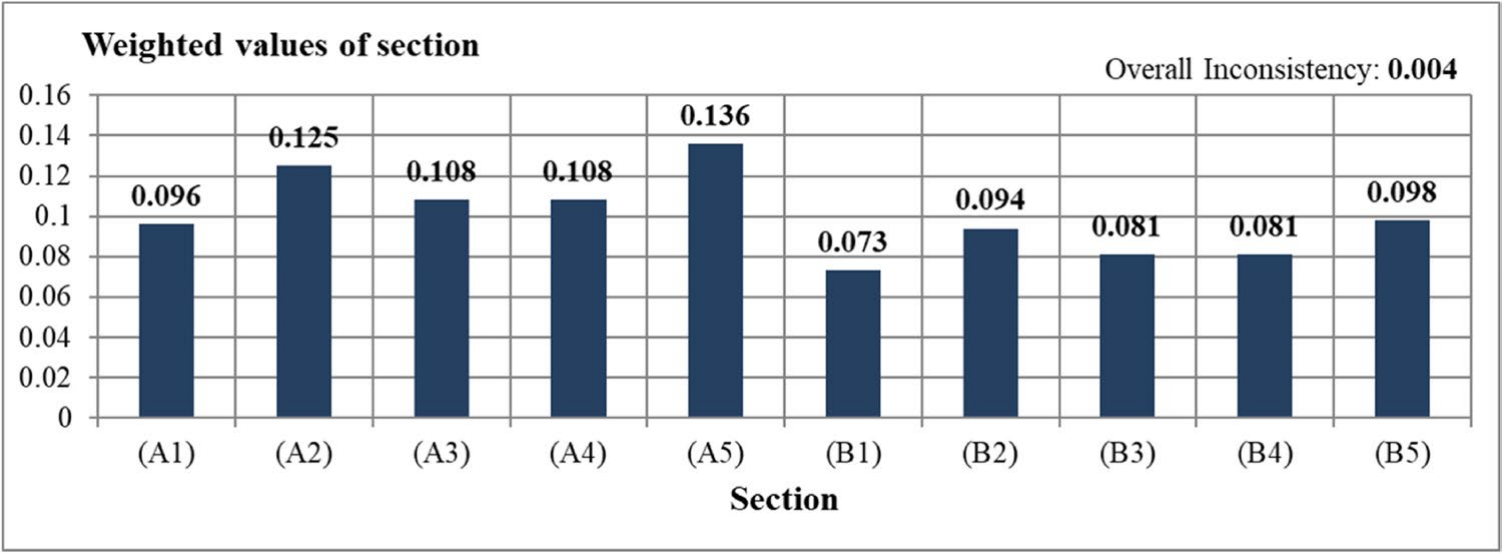
Weighted values of sections recommended for sustainable campuses

The sections under the planting landscape components from very high to very low were respectively A5: transportation (0.136) and A1: local environment (0.096). The order among the structural landscape components from very high to very low were respectively B5: transportation (0.098) and B1: local environment (0.073) (Fig. 5). In the AHP analysis performed between sections, the CTR ratio was 0.004. This result (A5) indicated that the transportation criterion was the highest priority criterion among both planting (A) and structural (B) criteria, while the local environment: (B1) sections was the least priority criterion.

### Prioritization credits criteria of sustainable campus

**Fig. 6:**
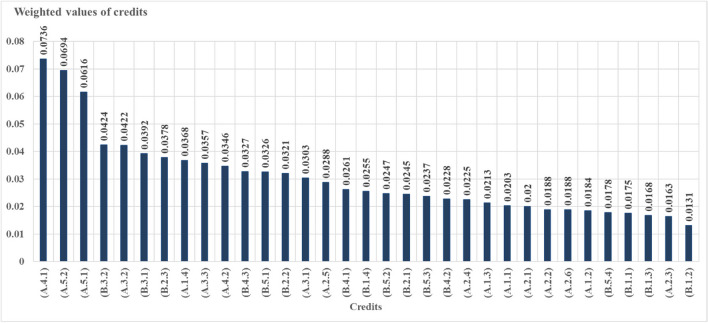
Weighted values of credits recommended for sustainable campuses

After determining the ranking among sections, AHP analysis was applied for credits that increased the original value of the study and were under sections, and they were compared in pairs. The most important credits under sections of local environment (A1) was A.1.4: increasing green areas (0.0368). The least important credits under this sections was found to be A.1.2: reducing CO_2_ emissions (0.0184). Since increasing green areas may reduce UHI and CO_2_ emissions, it was seen that this result was consistent. The most important credits under sections of water efficiency (A2) was A.2.5: use of natural plants (0.0288). Further, the least important credits under this sections was A.2.3: green roof (0.0163). The most important credits under sections of energy efficiency (A3) was determined to be A.3.2: green roof (0.0422). The least important credits under this sections was vertical garden (0.0303). The most important credits under sections of waste management (A4) was A.4.1: the use of plants as compost (0.0736). An important part of recycling might be achieved by using plants as compost. In addition, when all sustainable campus criteria were evaluated, the use of plants as compost was found to be the most important first criterion in sustainable campuses. In addition, when all sustainable campus criteria were evaluated, the use of plants as compost was found to be the most important first criterion in sustainable campuses. The most important credits under sections of transport (A5) was A.5.2: tree shape and location (0.0694). When all the sustainable campus criteria were evaluated, the tree shape and location of the plants was the second most important criterion in sustainable campuses. Besides, road afforestation was the third most important criterion (Fig. 6).

The most important credits under sections of local environment (B1) was B.1.4: the use of renewable materials (0.0255). This credit also revealed the importance of using rapidly renewable materials in nature. The least important credits under this sections was determined as B.1.2: construction activity pollution prevention (0.0131). The most important credits under sections of water efficiency (B2) was B.2.3: the use of permeable flooring material (0.0378). The least important credits under this sections was determined as B.2.1: gray water use (0.0245). The most important credits under sections of energy efficiency (B3) was B.3.2: the use of urban features elements that produce their own energy (0.0424). In addition, urban features that produces its own energy, which is under energy efficiency, was the fourth most important criterion among all sustainable campus criteria (Fig. 4). The least important credits under these sections was determined as the use of solar panels (0.0392). The most important credits under sections of waste management (B4) was B.4.3: On-site recycling of wastes such as paper, metal, and glass (0.0327). The least important credits under this sections was B.4.2: use of recycled flooring material (0.0228). The criterion with the highest score among credits under Transport (B5) was B.5.1: increasing access to public transportation (0.0326). The least important credits under this sections was determined as reducing the parking lot capacity (0.0178). Additionally, the CTR ratio among sections and credits was calculated and is shown in Table 2.

**Table 2:** Quantification of sections and credits of sustainable campus criteria

Criteria	Section value	CR	Credit	Credit value	CR
(A1)	0.096	0.004	(A.1.1)	0.0203	0.001
			(A.1.2)	0.0184	
			(A.1.3)	0.0213	
			(A.1.4)	0.0368	
(A2)	0.125		(A.2.1)	0.0200	0.04
			(A.2.2)	0.0188	
			(A.2.3)	0.0163	
			(A.2.4)	0.0225	
			(A.2.5)	0.0288	
			(A.2.6)	0.0188	
(A3)	0.108		(A.3.1)	0.0303	0.01
			(A.3.2)	0.0422	
			(A.3.3)	0.0357	
(A4)	0.108		(A.4.1)	0.0749	0.01
			(A.4.2)	0.0346	
(A5)	0.136		(A.5.1)	0.0616	0.01
			(A.5.2)	0.0694	
(B1)	0.073		(B.1.1)	0.0175	0.01
			(B.1.2)	0.0131	
			(B.1.3)	0.0168	
			(B.1.4)	0.0255	
(B2)	0.094		(B.2.1)	0.0245	0.05
			(B.2.2)	0.0321	
			(B.2.3)	0.0378	
(B3)	0.081		(B.3.1)	0.0392	0.01
			(B.3.2)	0.0424	
(B4)	0.081		(B.4.1)	0.0261	0.01
			(B.4.2)	0.0228	
			(B.4.3)	0.0327	
(B5)	0.098		(B.5.1)	0.0326	0.01
			(B.5.2)	0.0247	
			(B.5.3)	0.0237	
			(B.5.4)	0.0178	

*CR*, correction rate < 0.1

In the study area, when ranking in order importance among all credits among the criteria in sustainable campuses, the 3 most important credits were respectively A.4.1: using plant wastes as compost (0.0736), A.5.2: tree shapes and placements (0.0694), and A.5.1: road afforestation (0.0616) (Table 2). In addition, after these three titles, a serious break was observed (Fig. 5). This situation showed that there was a need to develop strategies for these three criteria in sustainable campus examples. The 3 least important credits were respectively B.1.2: construction activity pollution prevention (0.0131), A.2.3: green roof application under the subheading of water efficiency (0.0163), and B.1.3: use of certified wood (0.0168) (Table 2). According to this result, it is necessary to raise awareness about innovative uses.

## Discussion

Within the scope of the study, the survey conducted for theory- and practice-oriented landscapes in sustainable campuses was weighted by taking the AHP method into consideration. According to the AHP results, it was determined that the planting landscape components (A) were more important than the structural landscape components (B) in the ranking made among criteria. When the section under the criteria were examined, it was observed that the transport section (A5 and B5) for both structural and plant landscape components was more important for sustainable campuses. The local environment had the lowest score output for both categories. Sections and each of credits under it were evaluated separately in order of priorities. In addition, these results were classified as a practice and a theory under 5 sections. In this classification, reducing the UHI effect and CO_2_ emission reduction were included in the practice part. Other credits were included in the theory part of this study.

### Transport

**Fig. 7:**
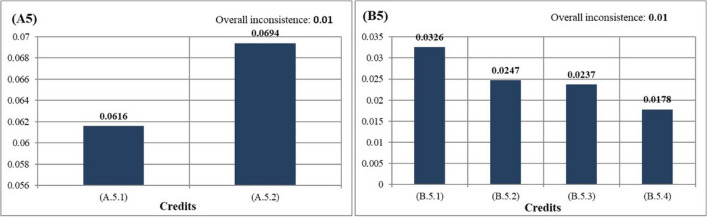
The priority ranking of credits under the planting (left) and structural (right) criteria and transport section

Evaluating credits under the transpor sections (A5) revealed that A.5.2: tree shape and placements was the most important criterion (Fig. 7) followed by tree shape and placements as the second most important criterion. According to Lachapelle et al. (2023), optimal tree shape and placements play a seminal role in reducing the urban heat island and mean radiant temperature (TMRT). According to the same study, tree clumps with high leaf area indices and high transpiration rates should be planted on wide streets, often with short buildings to help keep pedestrians cool in hot weather. In this context, *Platanus orientalis* species, which is naturally found in Bursa, has a wide crown, and provides shade, can be recommended for the open green areas in the study area. In new areas to be established, in addition to broad-leaved trees, small trees with little space between them should be preferred.

The second most important credits in the transport sections (A5), which is under the planting components, was A.5.1: road afforestation. Thanks to the road afforestation, reducing the UHI effect, less exposure to climatic extremes and less exposure of pedestrians to radiation might be achieved (Lachapelle et al. 2023). The use of broad-leaved trees should be encouraged on the impermeable roads passing near the educational buildings where thermal radiation is intense and leading to the parking lot areas on the Gorukle Campus.

Considering the ranking within the transport sections (B5), which was under the structural landscape components, it was seen that B.5.1: public transportation access was the most important criterion. This title is the criterion with the highest score in the sustainable areas category in the LEED certificate (UGB Council 2009). It has been surveyed that there is no direct public transportation access to some university buildings and dormitories in the study area. On the Gorukle Campus, access to university and dormitory buildings by public transportation should be increased and there should be a bus that makes a ring within the school. The least important credits was determined as B.5.4: reduction of parking lot capacity. Reducing parking lot capacity and promoting access using public transport, shared vehicles, and non-motorized vehicles, it is also included in globally accepted certificates such as LEED, BREEAM, and GreenMetric (GreenMetric 2022; BREEAM 2022; UGB Council 2009). However, in newly developing countries such as Turkey, the number of gasoline-powered vehicles is high due to the lack of knowledge of users on sustainable transport. This leads to the need for parking lot area. In this context, in the parking lot areas planned to be built on the Gorukle Campus in the future, the Parking Regulations (RTON 2018) Appendix. 1 should be taken into account. In these parking lot areas, parking lot areas should be provided for shared vehicles and hybrid vehicles. In addition, bicycle paths should be proposed in the area and all areas should be accessed by bicycle. Empty rooms of buildings with LEED certification can be used as showers and changing cabins (UGB Council 2009). In buildings where space is scarce, locker and shower cabins should be placed in areas close to the building entrances. Bicycle parking lots should be located in the immediate surrounding of the university entrances. Finally, parking lot areas for low-emission and fuel-efficient vehicles should be created on the Gorukle Campus. The upper part of these parking lot areas can be covered with solar panels. These panels, which convert the energy from the sun into electrical energy, can direct their energy to the storage areas in the parking lot.

### Water efficiency

**Fig. 8:**
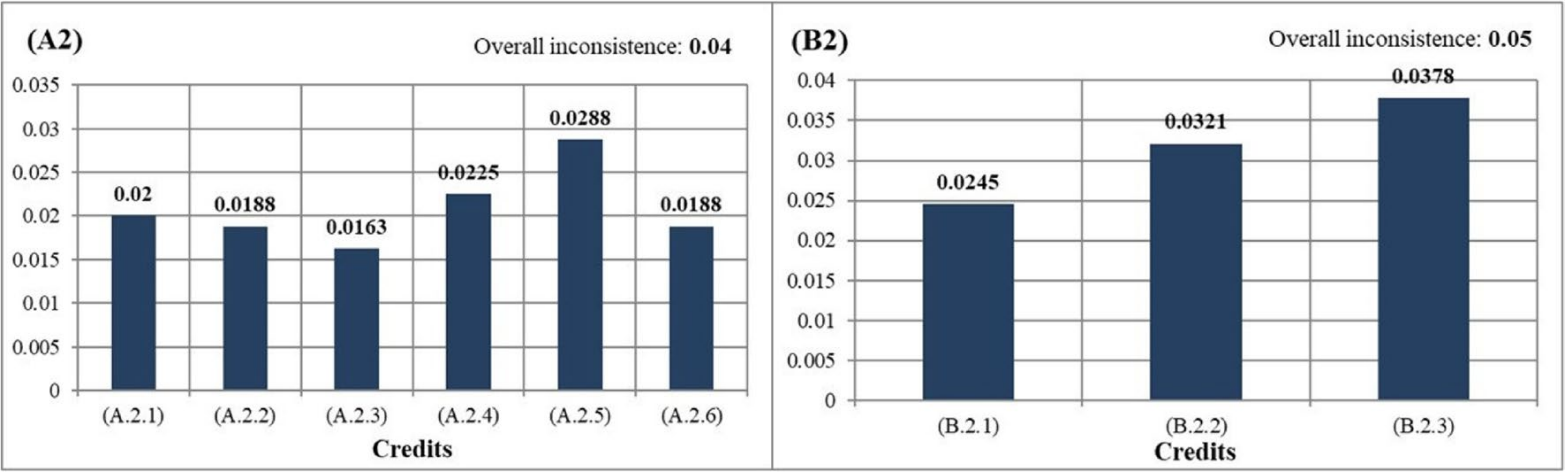
The priority ranking of credits under the planting (left) and structural (right) criteria and water efficiency section

In the ranking under the water efficiency (A2) category, which is under the planting landscape components (Fig. 8), A.2.5: the use of natural plants was determined as the most important criterion. With the use of natural plants, the amount of water consumption is reduced, and maintenance costs are saved (Amr et al. 2016). At the same time, the use of natural plant species in rain garden, xericape landscaping, and green roofs, which are sustainable practices, is encouraged. This shows that the result is consistent. Plant species naturally found in Bursa’s flora should be used in the planting and plant design works planned to be carried out in the Gorukle Campus in the future. The lowest score output under the water efficiency (A2) sections is A.2.3: green roof application. Green roofs on sustainable campuses includes different ecosystem services such as improved stormwater management, regulating building temperatures, reduced UHI, and providing habitat for living things (Song 2022). Despite all these advantages, the green roof application received the lowest value in credits of water efficiency. Considering these results, it can be predicted that experts made this choice by considering the costs of green roof implementation. The roofs allowed by the construction in the Gorukle Campus should be arranged as green roofs. The plants used in these green roofs should be selected from natural succulent plant species since succulent species have a high water holding capacity and savings can be achieved in indoor air conditioning costs in summer, for example, natural succulent plant species *Saxifraga sempervivum* and *Umbilicus erectus* can be used in Bursa.

Other applications with the least importance after green roofs in the study were xericape landscape and the reduction of grass areas. Grass areas are one of the areas with the highest water consumption (Kalayci Onac et al. 2021). In Las Vegas, a program that replaces grass, dry plants, and xeriscape groundcovers to conserve water has resulted in a savings of about 1/3 in water consumption (Vickers 2006). A similar study was conducted in Las Vegas within the scope of xeriscape, and in this study, it was observed that single-family houses use 76% less water than the others with xeriscape application (Hilaire et al. 2008). In the allocation of grass areas planned to be built in the future in the study area, natural, low water consumption, and drought-resistant *Festuca arundinacea*, *Phyla canescens*, and *Poa pratensis* species should be used.

Regarding the water efficiency category under the structural landscape components, it is seen that the use of permeable flooring material is chosen as the most important criterion (Fig. 8). There is an impermeable surface of 11.4 ha on the Gorukle Campus. Groundwater recharge can be provided with a permeable flooring material. It has also been proven by studies that there are some benefits such as water purification, reducing the UHI effect and recycling waste materials (Guan et al. 2021). Impermeable flooring materials should be replaced with permeable flooring materials in future applications in Gorukle Campus.

### Energy efficiency

**Fig. 9:**
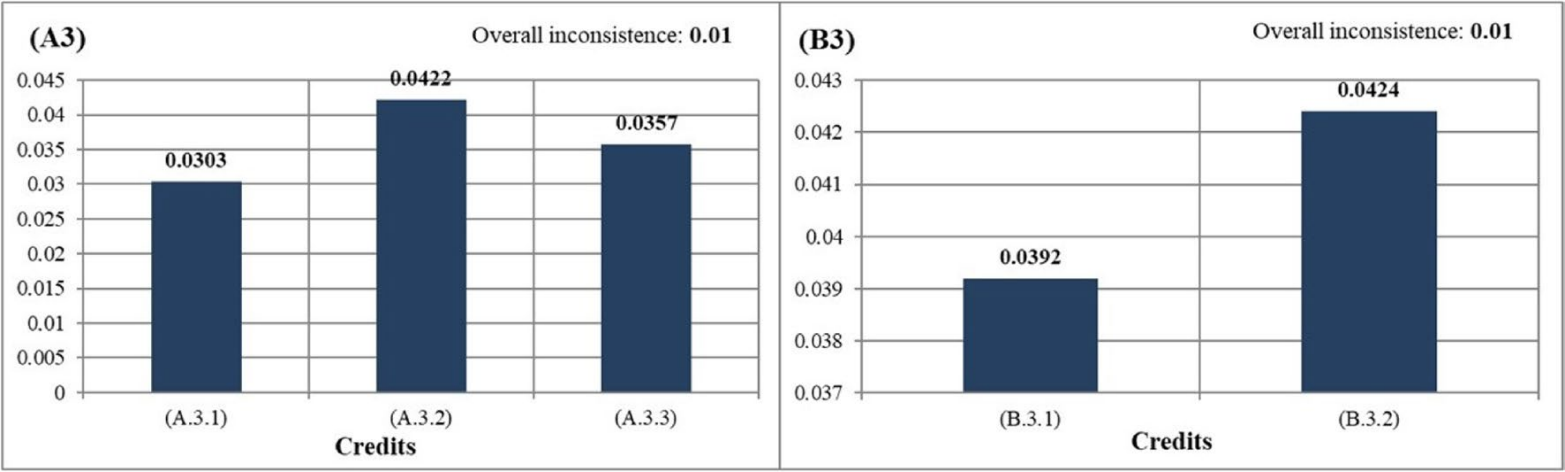
The priority ranking of credits under the planting (left) and structural (right) criteria and energy efficiency section

In the ranking under the energy efficiency (A3) category, which is under the planting landscape components, A.3.2: green roof application was determined as the most important criterion (Fig. 9). Green roofs are considered a sustainable practice in terms of providing energy savings by covering the exterior of a building with vegetation (Kalayci Onac et al. 2021). The green roof application under water efficiency (A2) is the penultimate title according to the general ranking among the sustainable campus criteria. However, the green roof application in the energy category (A3) ranks fifth among all criteria. This situation creates a contradiction. This result shows that the opinion of the experts that the green roof application is only used for energy saving purposes is dominant. However, green roofs are also important in terms of water conservation. In this context, awareness-raising activities should be carried out on green roofs.

The second most important credits under the energy category is A.3.3: wind curtain. Numerous studies have confirmed that using vegetation can significantly save cooling and heating energy (Zhu et al. 2022). For example, for a long-term windbreak, *Picea* spp., *Abies* spp., and *Thuja* spp. species that protect their sub-branches can be used. In the field survey made at the Gorukle Campus, there are many coniferous species that can be used as windbreaks, such as *Pinus pinea*, *Pinus nigra*, *Pinus brutia*, and *Cupressus* sp. These types should be used in the immediate surrounding of the education buildings on the Gorukle Campus. The least important credits in the energy sections, which is under the planting landscape components, was A.3.1: vertical gardening. Vertical gardens planned to be built in the future on Gorukle Campus should be placed on the façades where the prevailing wind is present. In this way, energy consumption costs in the building can be reduced (Perez et al. 2011). At the same time, Leaf Area Index (LAI) should be considered in plant selection. According to a study by Stav and Lawson (2012), increasing the leaf from 6 to 8 cm results in a dramatic increase in energy savings from 2 to 18%. In this context, *Hedera helix* type, which is naturally found in Bursa, should be recommended for building facades.

The most important credits in the energy category, which is one of the structural landscape components, was B.3.2: urban furniture that produces their own energy (Fig. 9). The lighting element used in England, one of the pioneers of smart urbanization in the world, produces its own energy (Ermiş and Karatekin 2019). On the Gorukle Campus, urban furniture that generates their own energy (benches, trash cans, etc.) should be used. A.3.1, which has a lower score than urban furniture that produces their own energy: the use of solar panels is the sixth most important criterion according to the overall ranking among the sustainable campus criteria. The use of solar panels is supported by experts, especially in Bursa, where the sunshine duration is low when compared to regions such as the southeast and central Anatolia of Turkey (RTMENR 2022). In this context, the most suitable areas for solar panels should be selected by making suitability analysis in Gorukle Campus. Solar panels should be chosen from architectural devices with the lowest solar reflectance index. A similar practice exists in CASBEE, LEED, BREEAM, and DGNB certifications.

### Waste management

**Fig. 10:**
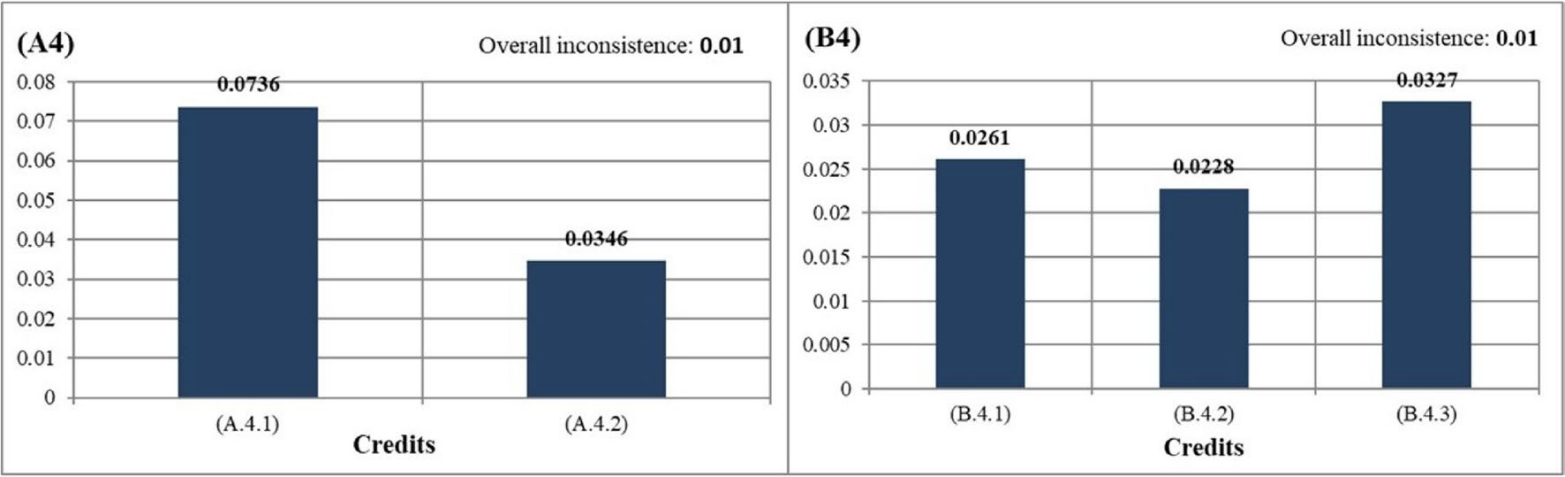
The priority ranking of credits under the planting (left) and structural (right) criteria and waste manegement section

Waste management sections ranked third as both structural (B4) and planting (A4). This section had the same coefficients as the energy sun criteria. However, the weight of the waste management (A4) category under the planting landscape components is higher than the waste management (B4) category under the structural landscape components. The most important credits of the waste management category (A4) was A.4.1: using plant wastes as compost. This credits is the most important criterion according to the overall ranking among all sustainable campus criteria (Fig. 10). This criterion has been determined as the first priority criterion by the students, academic staff, and university staff using the campus. In Marrakech, Morocco, green spaces and date palms account for 38% of urban waste. Treating this waste by composting will provide organic soil reclamation that can be used in the city’s gardens and reduce the imported peat expenditures (El Ouaqoudi et al. 2015). Approximately 1050 ha of the Gorukle Campus consists of forest, agricultural land, open green space, pastures, orchards, and natural plant areas. Planting wastes in these areas should be used as compost. Mulch is a material that keeps the soil moist, limits weed growth, and prevents lawn mowers and scythes from damaging the tree (Fitzgerald and Ries 1997). On the Gorukle Campus, grass areas should be reduced and mulching should be recommended as an alternative to turf.

The most important credits under the waste management (B4) sections was B.4.3: on-site recycling of wastes such as paper, metal, and glass. Waste reduction and recycling activities are the most popular campus greening programs (Kurtaslan 2020). Suitability analysis should be made in the study area and a compost processing facility should be proposed. In the compost processing plant, wood, twigs, leaves, and kitchen and grass wastes should be turned into compost. This compost should be used as fertilizer to be given to the plants. B.4.2 with the lowest score output under this category is the use of recycled flooring material. Gayarre et al. (2017) brought up the production of flooring materials in accordance with European standards by recycling ceramic and concrete wastes. In this context, the Fine Arts Department and the Civil Engineering Department located on the Gorukle Campus should take an active role in the recycling and use of rubble residues. In addition, the building materials of all urban furniture used in the area should be redesigned by considering recyclable and naturally site-specific materials.

### Local environment

**Fig. 11:**
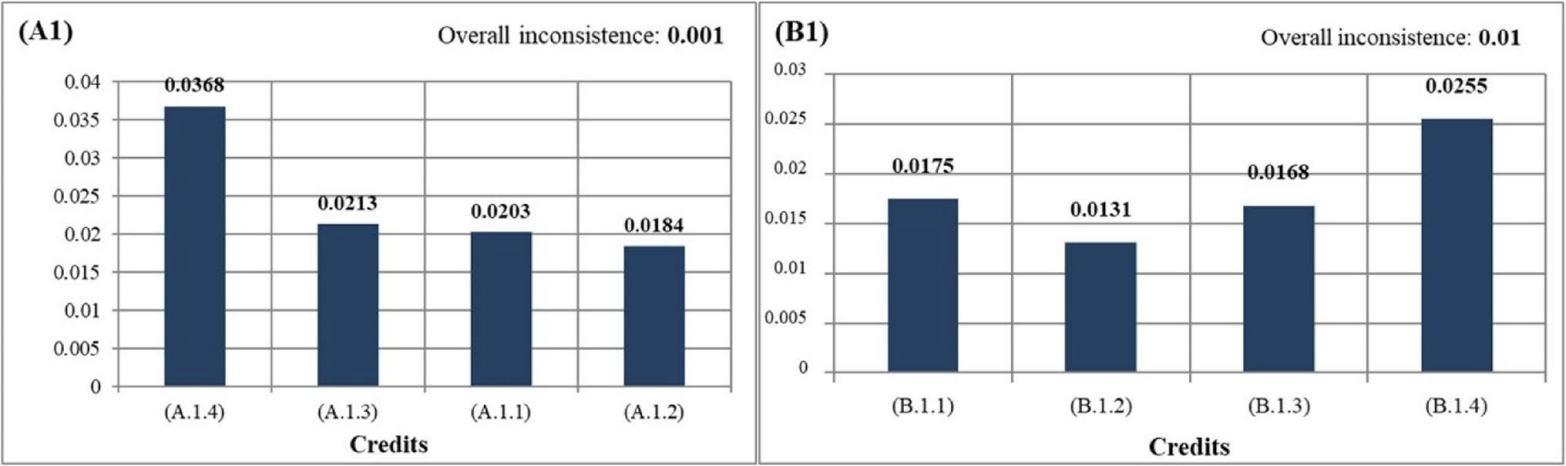
The priority ranking of credits under the planting (left) and structural (right) criteria and local environment section

The local environment category (A1 and B1) is the least important sections among both the planting landscape (A) and the structural landscape (B) components. Although many planting and structural credits under this sections are important, they have low score output in the survey results. In the ranking under the local environment category (A1), which is under the planting landscape components, A.1.4: increasing green areas was determined as the most important criterion for sustainable campuses (Fig. 11). Looking at the other credits under the local environment in the study area, it is seen that this data is quite consistent since with increasing green areas, the UHI effect and CO_2_ emissions can be reduced (Chen and You 2020). Green areas should be protected and green strategies (green infrastructure, green road) should be developed to increase green areas in the future works planned to be carried out on the Gorukle Campus. The second important credits under the local environment, A.1.3: development outside protected areas, is also included in the LEED certificate (UGB Council 2009). This credits argues that development should be provided at certain distances from wetlands, outside of fertile lands, and in areas where there is no living creature in the threat or danger category. For the university annex building or other structures planned to be built in the future on the Gorukle Campus, settlements should be developed outside the ecologically special and protected areas.

Considering the ranking under the local environment category under the structural landscape components (B1), B.4.2: the use of rapidly renewable materials was chosen as the most important criterion (Fig. 11). Renewable building materials and products are typically derived from crops harvested over a 10-year cycle or less (UGB Council 2009). Rapidly renewable materials should be used in the material selection of all equipment elements used in the Gorukle Campus. B.1.2: construction activity pollution prevention credits, which has the lowest score value under the local environment, received the lowest value in the questionnaires, although it is an important criterion in sustainable campuses since the pollution during construction harms both human and plant material, which causes the plant material to fail to fulfill its vital functions after a while. The second most important credits under this subcriterion is B.1.1: the use of regional flooring material. Naturally found in Bursa within the scope of permeable flooring material in the study area, materials such as Uludağ granite, Gemlik diabase, travertine, and marble should be used. In addition, wastes generated after the destruction of structural areas can be used in flooring materials used in the area. In addition, construction should not be developed on polluted areas in the area. Finally, the material of flooring or reinforcement elements whose structural material is planned to be wood in the future should be selected from products certified in accordance with the principles and criteria of the Forest Stewardship Council (UGB Council 2009).

Some limitations might be ascribed to this study. For example, due to the difficulty in accessing the Gorukle Campus and the limited time frame, most of the questionnaires were conducted online. At the same time, the awareness of the students and the experts who answered the questionnaires about the sustainable campus should be increased. At this point, collective studies should be carried out with relevant academicians and private companies that are experts in their fields, and long-term sustainable campus development plans should be prepared. In order to increase the acceptability of the study. In addition to the Gorukle Campus students, other university students should also be included in the questionnaire. In addition, the increase in the number of participants may contribute to the increase in the acceptability of the research. In other studies to be done, it should be examined in the social dimension, which is an important criterion in terms of sustainability. The most important limitation of the study is the pairwise comparison of too many criteria. An increase in the number of questions increases the time to answer the questionnaire. This may reduce the sensitivity of the study. In this context, it is recommended to use an innovative MCDM, best and worst method (BWM), in order to reduce the number of questions in future studies where the criteria are high. In particular, the technical details among the criteria strengthen the understanding of the problem by the participants. In this context, it is recommended to include workshops or small explanatory notes for preliminary information on the subject.

## Conclusion

The sustainable campus criteria for the Gorukle Campus are listed as “planting landscape components and structural landscape components” according to their importance among criteria. Among the criteria, it was revealed as a result of AHP that vegetative landscape components are more important than structural landscape components. The order of importance among sections under criteria was listed as “transportation, water efficient, energy efficient, waste management, and local environmental conditions”. In addition, a hierarchy was established among credits under sections that increased the original value of the study, and the importance was ranked, and the use of plants as compost in open green areas was determined as the most important criterion. All these results indicate that planning and design strategies for a “sustainable campus” in Gorukle Campus should be developed by focusing on these criteria.

In order to ensure sustainability in Gorukle Campus, holistic urban planning and design strategies taking into account the priorities of legal regulations and sustainable campus criteria should be determined. In this context, there is a need for a working process with the university, relevant ministries, local governments, experts, campus student societies, and other stakeholders. As a result, the methodology and findings of this research integrated the AHP method with participatory approach, and determined the quantitative sustainable campus criteria. Thus, this study, which was unique in its type, can set an exemplary quantitative model for the sustainable campus of Turkey and other countries.

## Data Availability

The data of the article can be provided, if it is requested by the authors.
